# Comparative Outcomes in Metastatic Spinal Cord Compression and Femoral Metastatic Disease: Distinct Clinical Entities with Divergent Prognoses?

**DOI:** 10.3390/medicina61081390

**Published:** 2025-07-31

**Authors:** Oded Hershkovich, Mojahed Sakhnini, Eyal Ramu, Boaz Liberman, Alon Friedlander, Raphael Lotan

**Affiliations:** 1 Department of Orthopedic Surgery, Wolfson Medical Center, Ha-Lokhamim St. 62, Holon 5822012, Israel; dr.lotan@gmail.com; 2 Gray Faculty of Medical and Health Sciences, Tel Aviv University, Tel Aviv 6997801, Israel; 3 Department of Orthopedic Surgery, Sheba Medical Center, Tel Aviv University, Tel Aviv 6997801, Israel

**Keywords:** cord, compression, femoral, metastases, mortality

## Abstract

*Background and Objectives*: Acute metastatic cord compression (AMSCC) and femoral impending/pathological fracture negatively impact a patient’s quality of life, morbidity and survival, and are considered significant life events. This study aims to compare AMSCC and FMD as distinct yet overlapping metastatic orthopedic emergencies, addressing whether they represent sequential disease stages or distinct patient subpopulations—an analysis critical for prognosis and treatment planning. *Materials and Methods*: Records of all patients who underwent surgery for a femoral metastatic disease (FMD) over a decade (2004–2015) and patients who were treated for acute metastatic spinal compression (AMSCC) (2007–2017) were retrieved. There were no patients lost to follow-up. *Results*: The treatment cohorts were similar in terms of age, gender, tumour origin, and the number of spinal metastases. Fifty-four patients were diagnosed with AMSCC. Following treatment, the Frankel muscle grading improved by 0.5 ± 0.8 grades. Two hundred and eighteen patients underwent surgical intervention for FMD. Seventy percent of femoral metastases were located in the femoral neck and trochanteric area. Impending fractures accounted for 52% of the cohort. The FMD cohort, including impending and pathological fractures, was similar to the AMSCC cohort in terms of age and the time interval between cancer diagnosis and surgery (56.7 ± 74.2 vs. 51.6 ± 69.6, respectively, *p* = 0.646). The Karnofsky functional score was higher for the FMD cohort (63.3 ± 16.2) than for the AMSCC cohort (48.5 ± 19.5; *p* < 0.001). The mean survival time for the FMD cohort was double that of the AMSCC, at 18.4 ± 23.5 months versus 9.1 ± 13.6 months, respectively (*p* = 0.006). *Conclusions*: In conclusion, this study is novel in proposing that FMD and AMSCC are distinct clinical entities, differing in their impact on patient function and, most importantly, on patient survival.

## 1. Introduction

The skeleton is the third most common site for metastatic involvement after the lung and liver. Prostate, breast, lung, renal and thyroid carcinomas account for 80% of all skeletal metastasis. The femur is the most affected site of long bones [1,2], while acute metastatic cord compression (AMSCC) is estimated to occur in up to 10% of patients with cancer [3,4]. Both entities negatively impact a patient’s quality of life, morbidity and survival and are considered significant life events for the patient and treating physician [5,6]. With improved oncological treatment, prolonged patient survival leads to an increased prevalence of metastatic osseous disease and eventually to impending or pathological fracture and AMSCC [7]. The impact of metastatic femoral and impending fractures has been studied, and fracture-preventing surgery is warranted to overcome potential fracture complications, such as deep vein thromboembolism, pneumonia, and urinary tract infections [8,9]. Several authors have extensively investigated potential prognostic factors [7,10,11,12,13,14,15,16]. Different predictive models with scoring systems for patient survival were suggested; however, estimating the actual patient’s survival in months or years with a metastatic bone disease remains challenging. Different factors play a role when considering long-bone metastatic disease or spinal involvement. The primary tumour origin is regarded as one of the most predictive prognostic survival factors [17,18,19]. Other significant factors are the Karnofsky functional score, visceral metastases, the hemoglobin count, and the number of metastases (metastasis load). Diagnosis of a pathological fracture signalling poor prognosis is still controversial; while some studies did not find a statistical correlation [7,20,21,22], others found worse outcomes if a pathological fracture occurred [23,24,25]. Other suggested factors include the time interval between cancer diagnosis and metastases occurrence (more than three years) and the patient’s conservative and surgical treatments [20,26,27].

AMSCC and FMD are two significant orthopedic metastatic challenges frequently encountered in orthopedic oncology, both of which require urgent intervention and shared multidisciplinary management. Both entities have been extensively studied regarding treatment protocols and their impact on functional outcomes and survival. Although each entity was studied individually, there is no cross-sectional data evaluation; are these the same patients at the same disease stages? Is this a natural evolution of the metastatic disease, or are we treating a different subpopulation of patients? This study aims to compare the prognostic weight of AMSCC and FMD in real-world orthopedic oncology practice.

## 2. Materials and Methods

A retrospective review was conducted of all patients who underwent surgical treatment for femoral metastatic disease (FMD) and acute metastatic spinal cord compression (AMSCC) at our institution. The FMD cohort included patients operated between January 2004 and December 2015, while the AMSCC cohort comprised patients treated between January 2007 and December 2017. All relevant clinical data were retrieved from institutional electronic medical records and archived patient files. Patients were followed until the time of death or for a minimum of 12 months following their recruitment into the study, whichever occurred first.

Data collection included demographic information, oncological history, pathological reports, surgical records, radiation therapy documentation, outpatient clinical follow-up notes, and imaging studies. Preoperative and postoperative imaging assessments encompassed conventional radiographs, computed tomography (CT) scans, bone scintigraphy, and positron emission tomography-computed tomography (PET-CT) where available. Mortality status and the exact date of death were confirmed through official records provided by the Ministry of Interior.

In the FMD cohort, patients presenting with impending pathological fractures of the femur were assessed using the Mirels classification system, which evaluates the risk of fracture based on lesion site, lesion size, radiographic appearance, and pain severity. Surgical intervention, either internal fixation or endoprosthetic arthroplasty, was indicated for patients with an established pathological fracture or an impending fracture with a Mirels score exceeding 7. The specific surgical approach was determined by the attending orthopedic oncologist based on the anatomical location of the lesion, the extent of bony destruction, and the patient’s overall clinical status.

For patients with AMSCC, diagnosis was established through magnetic resonance imaging (MRI) of the entire spine. Following radiological confirmation, treatment decisions were made by a multidisciplinary team, including a neurosurgeon or orthopedic spine surgeon and a radiation oncologist. Treatment strategies consisted of surgical decompression with or without stabilization, followed by adjuvant radiotherapy, or primary radiotherapy alone in patients deemed unsuitable for surgical intervention. The radiotherapy protocol was standardized, with patients receiving a total dose of 30 Gray (Gy) delivered in ten fractions using three-dimensional conformal radiation techniques. The first fraction of radiotherapy was administered within 72 h of AMSCC diagnosis to ensure timely decompression of the spinal cord.

Exclusion criteria encompassed patients who underwent surgical intervention for FMD or AMSCC at external medical centres, as well as those diagnosed with primary bone or soft tissue sarcomas. In cases of bilateral femoral metastatic involvement, the first surgery was included in the analysis, while further surgery was excluded from the analysis to avoid duplication of outcomes. There were no instances of loss to follow-up within the study population.

### Statistical Analysis

All statistical analyses were performed using IBM SPSS Statistics software, version 23.0 (IBM Corp., Armonk, NY, USA). Standard descriptive statistics were applied to summarize the study population characteristics, perioperative complication rates, and survival outcomes. Categorical variables are presented as absolute frequencies and corresponding percentages, while continuous variables are reported as mean values with standard deviations, provided that the data demonstrated a normal distribution.

The assumption of normality for continuous variables was assessed using the Shapiro–Wilk test. In cases where data deviated from a normal distribution, appropriate non-parametric alternatives were considered; however, for the purposes of the primary analyses described herein, only variables meeting normality assumptions were subjected to parametric testing.

Comparative analyses were conducted to evaluate differences in patient characteristics, anatomical sites of metastatic lesions, and surgical modalities. Continuous variables were compared across groups using one-way analysis of variance (ANOVA). When statistically significant differences were detected by ANOVA, post hoc multiple comparisons were performed using the Tukey–Kramer method to identify specific intergroup differences. For categorical variables, including systemic and local complication rates within 30 days postoperatively, the Fisher exact test was employed due to its accuracy in analyzing categorical data, particularly for small sample sizes.

Survival analysis was performed to assess postoperative outcomes. Follow-up time was defined as the interval from the date of surgery to the occurrence of death or the end of the follow-up period (censoring). Kaplan–Meier survival curves were plotted to estimate overall survival probabilities over time. Differences in survival distributions between groups were evaluated using the log-rank test (Mantel–Cox test). A two-sided *p*-value of 0.05 or less was considered indicative of statistical significance for all analyses.

The study received no external funding. Ethical approval was obtained from the institutional review board prior to data collection. Given the retrospective design of the study and the use of de-identified data, the requirement for informed consent was waived. All study procedures were conducted in accordance with institutional guidelines, the Declaration of Helsinki, and other relevant ethical standards.

## 3. Results

A total of 271 patients were included in the present study. Of these, 217 patients underwent surgical intervention for femoral metastatic disease (FMD), and 54 patients were diagnosed with acute metastatic spinal cord compression (AMSCC). The study cohort did not have patients with concomitant AMSCC and FMD.

### 3.1. AMSCC Cohort Characteristics

The AMSCC cohort included 54 patients who presented with varying degrees of neurological impairment, ranging from complete paraplegia to partial paraparesis. The mean age at presentation was 60.3 ± 15.1 years. The time interval between initial cancer diagnosis and AMSCC presentation was 4.3 ± 5.8 months. Neurological status at presentation was graded according to the Frankel classification: 35.2% of patients were classified as Frankel D, 42.6% as Frankel C, and 27.7% as Frankel B or A.

Following diagnosis, 32 patients (59.3%) underwent urgent surgical decompression within 48 h of hospital admission, while the remaining 22 patients (40.7%) received radiotherapy alone. Baseline characteristics, including age, gender distribution, primary tumour origin, and the number of spinal metastases, were similar between the surgical and radiotherapy-only subgroups. Functional neurological status improved by an average of 0.5 ± 0.8 Frankel grades following treatment.

Primary tumours among AMSCC patients were predominantly breast carcinoma (24.5%), lung carcinoma (17.0%), multiple myeloma (13.2%), and renal cell carcinoma (5.7%), with other malignancies occurring at lower frequencies. Notably, 14.9% of AMSCC cases were attributed to tumours categorized as “other” (Table 1). Solitary spinal metastases were present in 16.7% of AMSCC patients. The mean Karnofsky Performance Status score for this cohort was 48.5 ± 19.5, reflecting significant functional impairment.

### 3.2. Femoral Metastatic Disease (FMD) Cohort Characteristics

A total of 217 patients underwent surgical treatment for femoral metastatic disease. The mean age was 63.3 ± 12.8 years, with female patients accounting for 70.5% of the cohort. The higher proportion of females is likely attributable to the predominance of breast carcinoma, which was the most frequent primary malignancy (36.2%), followed by multiple myeloma (20.2%), lung carcinoma (17.9%), renal cell carcinoma (7.3%), melanoma (3.7%), and prostate carcinoma (3.2%).

Eight patients (3.7%) were diagnosed with femoral metastases at the time of cancer diagnosis, while the remaining 209 patients developed femoral involvement during the disease course. Among these, the time from primary tumour diagnosis to surgical intervention for femoral metastasis was 56.7 ± 74.2 months. Seventy percent of femoral metastases were located in the proximal femur, specifically in the femoral neck and trochanteric region, irrespective of primary tumour type. Metastases to the distal femur were less common, occurring in only 5.5% of cases.

At the time of surgery, 52% of patients had impending fractures, defined as lesions with a Mirels score of 7 or higher, while the remaining 48% had completed pathological fractures exceeding Mirels classification thresholds. Notably, 59% of surgical interventions in the femoral neck region and 93% in the subtrochanteric region were performed for completed fractures, with the remainder undergoing prophylactic stabilization for impending fractures.

The majority of FMD patients (85%) had multiple skeletal metastases at the time of surgery, and 52% exhibited extraskeletal metastatic disease. Solitary femoral metastases were documented in 7.8% of cases. The Karnofsky Performance Status score in this cohort was 63.3 ± 16.2, significantly higher than in the AMSCC group (*p* < 0.001), indicating a comparatively better functional status among FMD patients.

### 3.3. Comparative Analysis Between FMD and AMSCC Cohorts

Patient age was comparable between the FMD and AMSCC cohorts (63.3 ± 12.8 years vs. 60.3 ± 15.1 years, respectively; *p* = 0.316). A female predominance was observed in the FMD cohort (70.5%) compared to the AMSCC group (42.6%), approaching statistical significance (*p* = 0.072). Primary tumour distribution was similar between groups (*p* = 0.192), with breast carcinoma, multiple myeloma, and lung carcinoma being the most common etiologies in both cohorts.

The time from cancer diagnosis to surgical intervention or AMSCC presentation did not differ significantly between groups (56.7 ± 74.2 months for FMD vs. 51.6 ± 69.6 months for AMSCC, *p* = 0.646). However, the extent of metastatic disease differed markedly. Spinal metastases were present in 51.8% of FMD patients and, by definition, in 100% of AMSCC patients. Conversely, 14.8% of AMSCC patients also harboured femoral metastases at the time of presentation.

The FMD cohort exhibited significantly more widespread skeletal disease, with an average of 1.8 ± 1.2 osseous metastases compared to 0.9 ± 1.1 in the AMSCC cohort (*p* < 0.001). The number of soft tissue metastases did not differ significantly (*p* = 0.128).

Solitary metastases were significantly more common in the AMSCC cohort (16.7%) than in the FMD cohort (7.8%, *p* < 0.05), suggesting a higher proportion of isolated spinal involvement among AMSCC patients.

### 3.4. Survival Analysis

**Figure 1:**
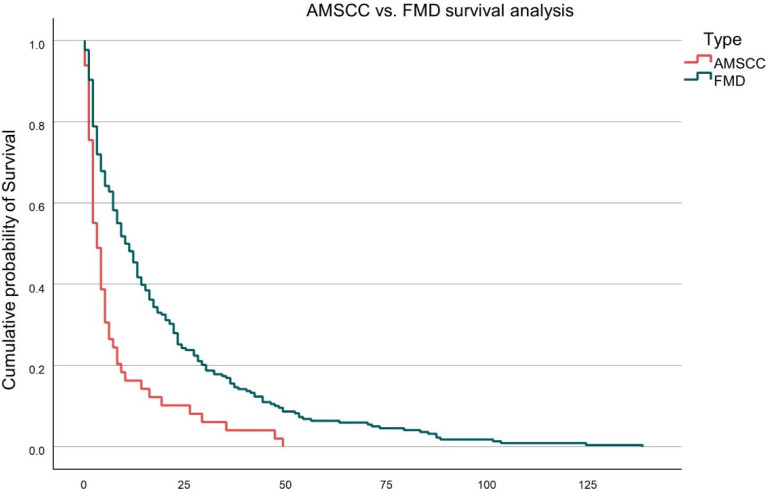
Kaplan–Meier survival plot FMD vs. AMSCC.

Overall survival was significantly inferior in the AMSCC cohort compared to FMD patients. The mean survival following intervention was 9.1 ± 13.6 months for AMSCC patients versus 18.4 ± 23.5 months for the FMD cohort (*p* = 0.006). This difference is illustrated in Figure 1, which depicts Kaplan–Meier survival curves for both groups. The AMSCC group exhibited a steeper decline in survival probability, with the majority of deaths occurring within the first 12 months following diagnosis.

**Figure 2:**
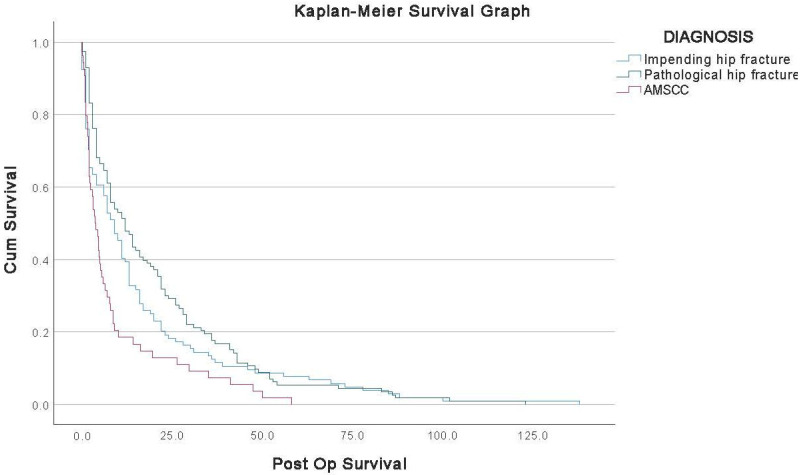
Kaplan–Meier survival plot: impending femoral fracture, femoral fracture and AMSCC.

Further stratification of survival within the FMD cohort revealed additional prognostic distinctions. As shown in Figure 2, patients with impending femoral fractures demonstrated the most favourable survival outcomes, followed by those with completed pathological femoral fractures. The AMSCC cohort consistently exhibited the poorest survival across all groups. The differences in survival curves were statistically significant (log-rank test, *p* < 0.01).

Cox regression analysis was performed, adjusting for the following parameters: age, sex, tumour type, Karnofsky Performance Score, number of osseous metastases, presence of extraskeletal disease, and solitary vs. multiple metastases. The Cox regression analysis confirms that AMSCC remains an independent predictor of decreased survival when adjusted for these variables. Specifically, compared to impending femoral fractures (reference group), the hazard ratio (HR) for AMSCC was 2.31 (95% CI: 1.49–3.58, *p* < 0.001), and for pathological femoral fractures was 1.56 (95% CI: 1.05–2.32, *p* = 0.028). These findings are consistent with the Kaplan–Meier survival analysis, as depicted in Figure 2.

## 4. Discussion

Femoral and metastatic spinal involvement are common illnesses, especially with improved surgical and oncological treatments. Femoral impending fractures, pathological fractures and metastatic spinal cord compressions are considered significant orthopedic morbidity and require timely treatment, especially AMSCC, which is regarded as urgent [5,10].

This study examined the FMD and AMSCC patients who presented to our medical centre over a decade. The cohorts were similar in age (*p* = 0.316) and tumour origin (*p* = 0.192); the most common tumours to metastasize the spine and femur were carcinoma of the breast, followed by Multiple Myeloma, Lung, renal cell, melanoma, and prostate (Table 1). On presentation, FMD patients had spinal metastases but no spinal cord compression or impending spinal fractures, and AMSCC patients had femoral metastases without impending fracture or pathological fracture (Table 1). Since both cohorts have femora and spinal involvement at presentation, it is essential to discern the significant metastatic manifestation since it affects survival.

We found no differences between cohorts regarding the time from cancer diagnosis to surgery, with 56.7 ± 6.2 months for the FMD cohort and 51.6 ± 5.8 months for the AMSCC, respectively (*p* = 0.646). Both cohorts developed devastating metastatic disease more than four years after diagnosis. The number of skeletal metastases on presentation was twice as high for the FMD cohorts as for the AMSCC, *p* < 0.001 (Table 1). Extraskeletal metastatic involvement was similar between cohorts (Table 1).

As measured by the Karnofsky score, the patient’s functional status was significantly lower in the AMSCC cohort compared to the FMD, at 48.5 versus 63.3, respectively, *p* < 0.001. Although the FMD cohort had significantly higher skeletal metastatic involvement, their functional status at presentation was significantly higher than that of the AMSCC cohort. This difference may be explained by the AMSCC cohort having a more aggressive tumour behaviour, although both cohorts had a similar tumoral origin [28]. We could not reaffirm this theory by calculating tumour aggressiveness, such as by the Katagiri classification or the Pathologic Fracture Mortality Index. Another possible explanation is the impact of the anatomical location of the tumour on the patient’s function.

The median survival time was half that of the AMSCC cohort compared to the FMD, 9.1 ± 13.6 months vs. 18.4 ± 23.5 months, respectively, *p* = 0.006 (Table 1). In a subanalysis of AMSCC, impending and actual femoral fracture survival, the AMSCC had the worst survival rate, followed by femoral metastatic fracture, with impending fractures having the best survival rate (Figure 2) (*p* <0.01). This data suggests that although the FMD cohort has a higher metastatic burden than AMSCC, it still carries a better prognosis. On the other hand, AMSCC appears to be a more aggressive tumoral manifestation that tends to reduce a patient’s function and survival compared to FMD. Paradoxically, but in line with tumour behaviour, solitary metastatic disease, which is typically associated with a better prognosis, was more prevalent in the AMSCC cohort than at the FMD, 16.7% vs. 7.8% (*p* < 0.05), respectively. However, the AMSCC cohort still had a worse prognosis, suggesting a more aggressive disease or the consequence of neurological deficits with immobility and infection sequela.

To address potential confounding variables and strengthen the validity of our survival comparisons, we conducted a multivariable Cox proportional hazards regression analysis. This model adjusted for critical covariates, including patient age, sex, tumour origin, Karnofsky Performance Score, number of osseous metastases, presence of extraskeletal disease, solitary versus multiple metastatic lesions, and treatment modality. After adjustment, AMSCC remained an independent predictor of worse survival compared to impending femoral fractures, with a hazard ratio (HR) of 2.31 (95% CI: 1.49–3.58, *p* < 0.001). Pathological femoral fractures also carried a significantly increased risk of mortality compared to impending fractures (HR = 1.56, 95% CI: 1.05–2.32, *p* = 0.028). These results confirm that the observed survival differences were not solely attributable to variations in baseline characteristics or metastatic burden. Instead, they reinforce the conclusion that AMSCC represents a more aggressive metastatic manifestation, associated with higher functional impairment and reduced overall survival, even when controlling for known prognostic factors.

The study’s limitations include its retrospective nature and the fact that cohorts were assessed in overlapping but not identical years. The female proportion in the cohort differed; however, a subanalysis within the cohorts did not reveal a statistically significant difference in gender-related survival. While our comparative analysis between AMSCC and FMD yields valuable insights into metastatic disease patterns, we acknowledge the conceptual limitations inherent in juxtaposing these two distinct clinical entities. AMSCC is a neurologic emergency driven by spinal cord compromise, often reflecting rapid local progression, whereas FMD generally represents a mechanical failure of load-bearing bone, developing over a longer time frame. Their differing anatomical, functional, and temporal characteristics pose challenges to direct comparison. However, we believe that examining these conditions side by side provides meaningful pragmatic insights.

AMSCC and FMD represent two urgent and impactful manifestations of skeletal metastases commonly encountered in orthopedic surgery. While both conditions have been the focus of extensive research individually—with well-established treatment protocols and documented effects on survival and function—the relationship between them remains unclear. To date, no cross-sectional studies have directly examined whether AMSCC and FMD affect the same patient population or reflect different stages in the progression of metastatic disease. Clarifying this relationship is essential, as it has direct implications for clinical decision-making, prognostic assessment, and the optimization of multidisciplinary care strategies.

The findings of this study carry important clinical implications for oncologic care planning. First, they suggest that not all metastatic skeletal events carry the same prognostic weight, even when arising from similar primary malignancies. Specifically, AMSCC appears to represent a more aggressive and life-limiting manifestation than FMD, as evidenced by the significantly lower Karnofsky scores and shorter survival times, despite fewer skeletal metastases on average. This information can assist clinicians in stratifying patients by risk and urgency: AMSCC should trigger expedited multidisciplinary evaluation, with input from neurosurgery and radiation oncology prioritized, given its more rapid impact on function and survival. In contrast, while FMD requires timely orthopedic intervention to prevent complications from fracture, these patients may have a broader therapeutic window and more opportunity for optimization prior to surgery. Moreover, understanding that AMSCC confers an independently worse prognosis—even after adjustment for known survival predictors—can improve communication with patients and families about expected outcomes and guide discussions about goals of care. The treatment protocol for AMSCC and FMD will not change with this study; however, understanding that these pathologies carry different prognoses and appear at different stages of metastatic disease is important in determining treatment strategy rather than tactics.

## 5. Conclusions

In conclusion, this study is novel in proposing that FMD and AMSCC are distinct clinical entities, differing in their impact on patient function and, most importantly, patient survival. AMSCC appears to represent a more aggressive form of metastatic disease than FMD. Since both FMD and AMSCC carry a reduced prognosis, they should be actively investigated by treating physicians in cancer patients, with timely consultation from a surgeon.

## Figures and Tables

**Table 1:** Baseline characteristics of AMSCC patients.

	Femoral Mets (217)	AMSCC (54)	*p*-Value
Mean age (y)	63.3 ± 12.8	60.3 ± 15.1	0.316
Female (%)	70.5	42.6	0.072
Primary tumours			0.192
Breast	36.2% (79)	24.5% (13)	
Multiple Myeloma	20.2% (44)	13.2% (7)	
Lung	17.9% (39)	17.0% (9)	
RCC	7.3% (16)	5.7% (3)	
Melanoma	3.7% (8)	5.7% (3)	
Prostate	3.2% (7)	7.6% (4)	
Colon	1.8% (4)	3.8% (2)	
Lymphoma	1.8% (4)	1.9% (1)	
TCC	1.4% (3)	3.8% (2)	
Thyroid	1.4% (3)	1.9% (1)	
Other	5.1% (11)	14.9% (8)	
Tumour diagnosis to intervention (m)	56.7 ± 6.2	51.6 ± 5.8	0.646
Spinal metastases	51.8%		<0.001
Femoral metastases		14.8%	
Osseous Metastases	1.8 ± 1.2	0.9 ± 1.1	<0.001
Soft Tissue Metastases	0.8 ± 1	1.1 ± 1.4	0.128
Solitary Metastasis	7.8%	16.7%	<0.05
Karnofsky score	63.3 ± 16.2	48.5 ± 19.5	<0.001
Average time to death (months)	18.4 ± 23.5	9.1 ± 13.6	0.006

## Data Availability

The datasets generated during and/or analyzed during the current study are available from the corresponding author upon reasonable request.
